# Targeting IL-6 in antibody-mediated kidney transplant rejection

**DOI:** 10.1093/ckj/sfaf108

**Published:** 2025-04-15

**Authors:** Mehmet Kanbay, Berk Mizrak, Sidar Copur, Ezgi N Alper, Sebahat Akgul, Alberto Ortiz, Caner Susal

**Affiliations:** Department of Medicine, Division of Nephrology, Koc University School of Medicine, Istanbul, Turkey; Department of Medicine, Koc University School of Medicine, Istanbul, Turkey; Department of Internal Medicine, Koc University School of Medicine, Istanbul, Turkey; Department of Medicine, Koc University School of Medicine, Istanbul, Turkey; Transplant Immunology Research Center of Excellence TIREX, Koç University Hospital, Istanbul, Turkey; Department of Medicine, Universidad Autonoma de Madrid and IIS-Fundacion Jimenez Diaz, Madrid, Spain; Transplant Immunology Research Center of Excellence TIREX, Koç University Hospital, Istanbul, Turkey

**Keywords:** antibody-mediated rejection, canakinumab, interleukin 6, kidney transplantation, tocilizumab

## Abstract

Interleukin (IL)-6 is a major pro-inflammatory cytokine and central regulator of innate and adaptive immune responses. Clinical trials testing antibodies against IL-6 or its receptors have demonstrated its involvement in the pathogenesis of several autoimmune and inflammatory disorders and in the systemic inflammation and anemia associated to kidney failure and also in kidney allograft rejection. Additionally, the anti-IL-6 receptor antibody tocilizumab and the anti-IL-6 antibody clazakizumab have been studied for the treatment of naïve as well as resistant antibody-mediated kidney allograft rejection with mixed results in observational studies and early clinical development. Following promising results with a clazakizumab in a phase 2 placebo-controlled trial, a large phase 3 trial (IMAGINE) was terminated in 2024 for futility at interim analysis. Investigator-initiated clinical development continues in a smaller phase 3 trial testing tocilizumab (INTERCEPT). In this viewpoint article, we evaluate the pathophysiology of IL-6 in antibody-mediated kidney allograft rejection along with the current status of the clinical development of IL-6 targeting therapies for antibody-mediated kidney allograft rejection episodes within the wider frame of IL-6 targeting therapies in kidney failure that are considered the major causes of graft loss in kidney transplantation.

## INTRODUCTION

Chronic kidney disease (CKD) represents a growing global health issue, which conveys an increased risk of cardiovascular disease and death that is not fully reversed by kidney replacement therapy in patients with kidney failure [1]. Although kidney transplantation represents the best kidney replacement therapy option, the outcomes of kidney transplantation should be improved and the treatment of antibody-mediated rejection (AMR) of kidney allografts remains a key unmet need.

**Figure 1:**
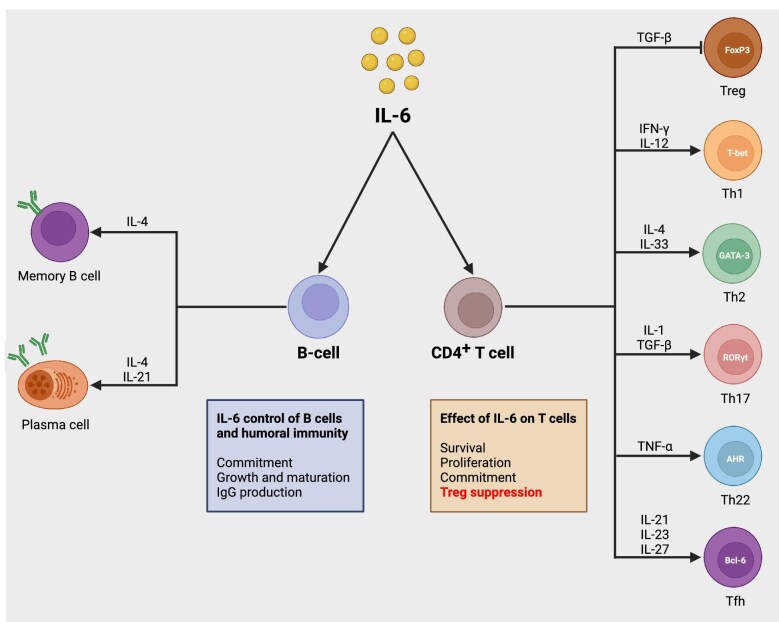
The role of interleukin (IL)-6 in the regulation of various immune pathways under physiological conditions. IL-6 has a pivotal role in the regulation of human immune system involving (i) regulation of B-cell differentiation, proliferation, and antibody production and (ii) upregulation of T-cell proliferation, differentiation, and survival along with the downregulation of regulatory T cells. FoxP3, Forkhead box P3; Th, T-helper cell; T-bet, T-box transcription factor TBX21; GATA-3, GATA-binding protein 3; RORγt, retinoic acid receptor-related orphan receptor gamma t; AHR, aryl hydrocarbon receptor, Tfh, T follicular helper cell; Bcl-6, B-cell lymphoma 6 protein; TGF-β, transforming growth factor beta; IFN-γ, interferon gamma; TNF-α, tumor necrosis factor alpha; IgG, immunoglobulin G.

Interleukin-6 (IL-6) is a key pleiotropic, pro-inflammatory cytokine produced by a variety of cell types, including lymphocytes, monocytes, and fibroblasts, [2] which participates in immune responses, inflammation, metabolic changes, and hematopoiesis [2]. IL-6 plays a central role in both innate and adaptive immunity including differentiation of B cells into antibody-producing plasma cells and differentiation of naïve T cells into T-helper 17 cells and T follicular helper cells [3] (Fig. 1). Through its involvement in both innate and adaptive immune responses, inflammation, and tissue injury, IL-6 plays a significant role also in kidney allograft rejection [3]. Indeed, early clinical data suggest that blocking IL-6 activity could be a promising strategy for the prevention and management of kidney allograft injury [3].

Several monoclonal antibodies in clinical use target either IL-6 itself or its receptor (IL-6R) [4]. Tocilizumab was the first IL-6 inhibiting agent that demonstrated efficacy and a good safety profile in treating human autoimmune diseases [5]. Additionally, the IL-6R-binding antibodies tocilizumab, sarilumab, and satralizumab and the IL-6-binding monoclonal antibodies clazakizumab, siltuximab, olokizumab, and ziltivekimab have been evaluated in clinical trials or are approved for clinical use (Table 1) [5]. Some of these antibodies (e.g. clazakizumab and ziltivekimab) have shown beneficial effects on inflammation and anemia and are in clinical development for cardiovascular protection in CKD and kidney failure [6, 7]. In this review, we evaluate the existing knowledge on IL-6 inhibitors and their impact on kidney transplant outcomes in AMR of kidney allografts.

**Table 1:** Anti-IL6 therapies for the management of treatment-naïve or treatment-resistant antibody-mediated kidney transplant rejection episodes.

Study	Study design	Participants (*n*)	Participant characteristics	Outcome
Tocilizumab				
Choi *et al.* 2017 [17]	Single-center open-label case study	36	mean age 45.9; male: 52.8%; mean eGFR 48.4; DSA+ 91.7%; mean DSAs 1.91	Tocilizumab stabilized eGFR (38.8 ± 10.4 ml/min/1.73 m² in adults) over 3.26 years, with graft and patient survival rates of 80% and 91% at 6 years, respectively. Biopsies showed significant reductions in g + ptc (*P* = .0175) and C4d (*P* = .0318) scores, alongside decreased iDSA levels at 24 months. Adverse events included infections (36%), cardiovascular events (8%), and hypogammaglobulinemia (22%), with 11.1% graft loss primarily due to therapy discontinuation.
Pottebaum *et al.* 2020 [25]	Single-center retrospective cohort study	7	mean age 42.9; male 42.8%	Renal function improved or stabilized in all patients (*n* = 7). Adverse events: CMV esophagitis (1), hypersensitivity reaction (1). Mixed or T-cell rejection occurred in three cases (6–24 months post-therapy).
Kumar *et al.* 2020 [26]	Single-center observational cohort study	10	mean age 43.3; male 60%; DSA+ 80%	Renal function showed no significant improvement (eGFR: 42 ± 18 to 37±24 ml/min/1.73 m²; *P* = .27), and the slope of eGFR decline remained unchanged (−0.14 ± 0.9 to −0.33 ± 1.1; *P* = .25). MVI (4.8 ± 1.4 to 4.2 ± 2.0; *P* = .39), AbMR scores (0.79 ± 0.17 to 0.78 ± 0.26; *P* = .86), and chronicity (2.5 ± 0.8 to 3.3 ± 1.7; *P* = .38) also showed no improvement. Patient survival was 90%, with one death from hip infection, while death-censored graft survival was 80%, with two graft losses due to recurrent infections.
Lavacca *et al.* 2020 [18]	Single-center open-label case study	15	mean age 38.3; male 80%; mean eGFR 45.1; DSA+ 100%	Stabilized eGFR and proteinuria, significant DSA reduction, improved microvascular inflammation, and absence of TG, C4d deposition, or IF/TA progression in follow-up biopsies.
Noble *et al.* 2021 [27]	Single-center retrospective study	44	mean age 43; male 60%	No significant change in eGFR (41.6 ± 17 vs. 43 ± 17 ml/min/1.73 m²; *P* = .102). Graft loss 6 (15%). Histological follow-up showed improved arteritis and IFTA inflammation but increased chronic glomerulopathy scores.
Clazakizumab				
Jordan *et al.* 2022 [32]	Single-center open-label observational study	10	mean age 51; male 70%; mean DSA MFI: 9 625	Significant eGFR decline prior to treatment (−24 months to baseline: 52.8 ± 14.6 to 38.1 ± 12.2 ml/min/1.73 m²; *P* = .03), but stabilized posttreatment (38.1 ± 20.3 at 24 months). Pre- and posttreatment biopsies showed reductions in g + ptc and C4d scores. DSA decreased in most patients. Minimal adverse events; two graft losses occurred in patients who discontinued therapy early.
Doberer *et al.* 2021 [33]	Phase II randomized pilot trial	20	mean age 34.2; male 50%; mean eGFR 39.3; mean uPCR 962; mean DSA MFI 11 708	Slower eGFR decline with clazakizumab compared to placebo during part A (−0.96 vs. −2.43 ml/min/1.73 m² per month; *P* = .04). In part B, patients switching from placebo to clazakizumab showed an improved eGFR slope, with no significant difference from those initially treated with clazakizumab significantly from patients initially allocated to clazakizumab.

MFI, mean fluorescence intensity; IF/TA, interstitial fibrosis/tubular atrophy; g, glomerulitis; ptc, peritubular capillaritis; ci, interstitial fibrosis; ct, tubular atrophy; TG, transplant glomerulopathy; uPCR, urinary protein-to-creatinine ratio (mg/g). Age expressed in years.

## ANTIBODY-MEDIATED KIDNEY ALLOGRAFT REJECTION

AMR is a cardinal cause of kidney allograft failure [8]. Although there have been considerable advances in understanding its molecular mechanisms, there is still no approved effective treatment for AMR [8]. To address this issue, expert panels have suggested off-label treatment strategies, which primarily focus on optimizing standard therapy protocols that include rituximab, plasma exchange, and intravenous immunoglobins (IVIG) [9]. Currently, numerous innovative strategies are being developed for the control of AMR, with one notable approach being the targeting of the IL-6 pathway to mitigate B-cell alloimmunity [10].

### IL-6 in antibody-mediated kidney allograft rejection

In addition to its general role in mediating and promoting inflammation, IL-6 plays a critical role in both innate and adaptive immune responses including activation of B cells and their differentiation into antibody-secreting plasma cells that produce donor-specific human leukocyte antigen (HLA) antibodies (DSA) among kidney transplant recipients [11, 12]. Consequently, inhibiting IL-6 could prevent as well as control AMR [10]. Over-production of IL-6 may also lead to chronic inflammation and neovascularization [11, 12]. Additionally, IL-6 is involved in activation of long-lived pro-inflammatory T-helper cells including follicular T-helper cells as well as inhibition of regulatory T cells (Tregs), further enhancing the pro-inflammatory signals [13].

### IL-6 targeting in antibody-mediated kidney allograft rejection

In animal studies, antibodies targeting IL-6R reduced the risk for graft-versus-host disease and allograft rejection [13, 14] and attenuate de novo DSA production and alloantibody recall responses [15, 16]. This effect is achieved through modulation of Tregs and effector cells in alloimmunized animals, showing a potential for favorable outcomes in prevention and treatment of AMR [15, 16] (Fig. 1).

**Figure 2:**
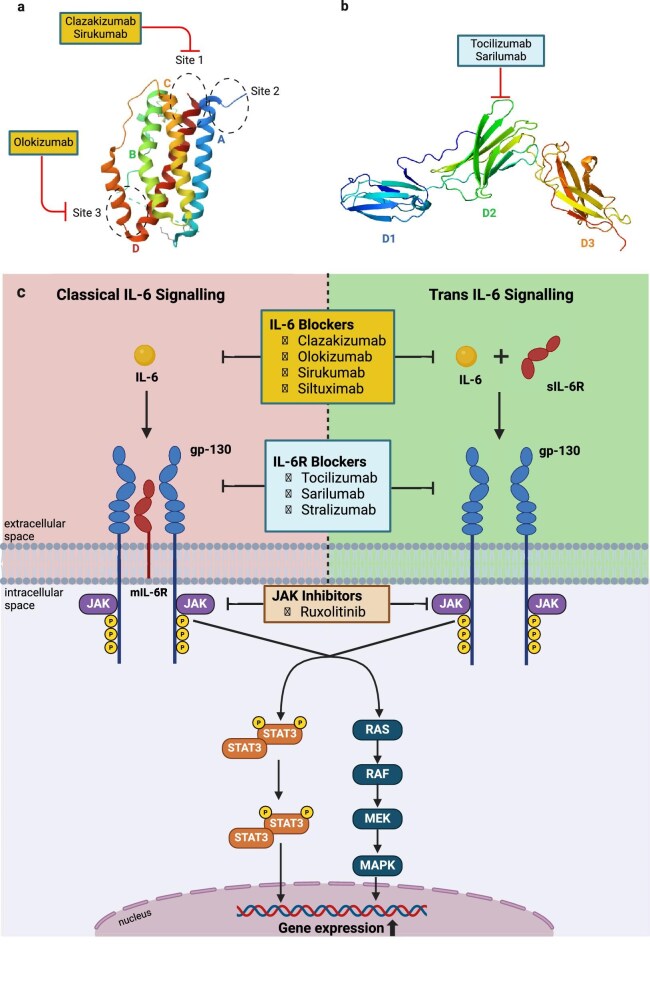
(a) Detailed structure of a IL-6 molecule and interaction with inhibitory antibodies. (b) Effect of IL-6 blockage by clazakizumab and tocilizumab on kidney transplant. (c) Classical and trans IL-6 signaling pathways and mechanisms of pharmacologic inhibition. mIL-6R, membrane-bound interleukin-6 receptor; sIL-6R, soluble interleukin-6 receptor; gp130, glycoprotein 130.

The use of IL-6 pathway inhibitors for AMR has been investigated in a limited number of clinical studies. Data from a few uncontrolled studies and individual case reports suggest that blocking the IL-6R with tocilizumab in patients with AMR can stabilize kidney function, adjust levels of DSA, and improve histological changes [17–20]. Despite these promising findings, a notable gap remains regarding the effectiveness of IL-6 pathway inhibition in treating chronic forms of AMR (cAMR), highlighting the need for a more thorough evaluation through controlled clinical trials (Fig. 2b). Currently, only tocilizumab and clazakizumab have been studied in kidney transplant recipients; while, sarilumab, siltuximab, satralizumab, olokizumab, and ziltivekimab have not been evaluated.

#### Tocilizumab

Tocilizumab is a humanized recombinant monoclonal antibody targeting soluble and membrane-bound IL-6 receptors, first approved as an orphan drug for the management of Castleman's disease [21, 22]. The use of tocilizumab is indicated to treat rheumatoid arthritis, juvenile idiopathic arthritis, Takayasu's arteritis, giant cell arteritis, polymyalgia rheumatica, and adult-onset Still disease [23].

##### Tocilizumab as rescue therapy for AMR

A single-center open-label clinical study studied 36 kidney transplant recipients with DSA-positive cAMR, with and without accompanying transplant glomerulopathy, who failed to respond to standard of care treatment under IVIG plus rituximab, with and without additional plasmapheresis. Maintenance immunosuppressive regimen in this study included tacrolimus, mycophenolate mofetil, and prednisolone for all participants. Tocilizumab (8 mg/kg/month) was administered for 6 to 25 months, resulting in allograft loss in only four patients (11.1%) after a median of 3.26 years following administration of the initial dose. Tocilizumab therapy also led to a stable estimated glomerular filtration rate (eGFR) course along with significant decline in DSA levels during the follow-up period of 3.26 years. Infectious adverse events were observed in 13 participants, five of which were cytomegalovirus infections. Additionally, thromboembolic events including stroke and non-ST segment elevation myocardial infarction occurred, however, not necessarily linked to tocilizumab therapy [17]. This study lacked a control group.

By contrast, a retrospective case series study involving nine kidney transplant recipients with treatment-refractory (IVIG, plasmapheresis, rituximab) cAMR who received monthly tocilizumab therapy compared to a historical cohort of 37 patients with similar characteristics did not observe differences in terms of eGFR decline (−4.0 ml/min/1.73 m^2^ versus −2.9 ml/min/1.73 m^2^; *P* = .18) or graft survival over 1 year. Histopathology revealed a stable glomerulopathy and a numerical (not statistically significant) decline in inflammation + tubulitis scores in the tocilizumab group (from 1.78 ± 1.40 to 0.57 ± 0.79, *P* = .07), while stable numbers were observed for historical controls (from 0.75 ± 1.25 to 1.0 ± 1.30, *P* = .47). Furthermore, interstitial fibrosis and tubular atrophy scores increased [24]. Few other studies with small sample size evaluating the potential role of tocilizumab therapy as a rescue therapy for cAMR patients have demonstrated contradictory outcomes [25, 26]. Thus, results from uncontrolled clinical observations were inconclusive regarding tocilizumab as rescue therapy for AMR. Nonetheless, it is important to acknowledge the fact that most patients included in such clinical trials evaluating the efficacy and safety of tocilizumab therapy among conventional therapy-refractory cAMR patients had histopathological evidence of advanced glomerulopathy, a chronic and most likely an irreversible lesion. Therefore, it is challenging and potentially misleading to interpret the efficacy of such therapeutic option among patients with high degree of irreversible histopathological lesions.

##### Tocilizumab as first-line therapy in cAMR

Tocilizumab therapy was also studied as a first-line therapeutic option in an uncontrolled small clinical trial involving 15 cAMR patients with advanced transplant glomerulopathy. eGFR and proteinuria remained stable and there was a significant decline in DSA titers during a median follow-up of 20.7 months. Protocol biopsies at month 6 observed an improvement in inflammation along without any progression of C4d deposition, transplant glomerulopathy, or tubular atrophy/interstitial fibrosis scores. Gene expression analysis demonstrated upregulation of TJP-1, a mediator of mTOR signaling pathway, of a member of intracellular zonula occludens, aldo-keto reductase family 1 member C3 (AKR1C3), a protein involved in inflammation and cellular differentiation, and of calcium/calmodulin dependent serine protein kinase. Bacterial infections were observed in five patients and promptly responded to anti-bacterial therapy. Additionally, interstitial lung disease was observed in two patients, and hypogammaglobulinemia in four patients [18]. This clinical study suggests that early, first-line tocilizumab administration can stabilize kidney injury in cAMR, as previous ineffective immunosuppressive therapy periods may underlie the possible irreversibility of cAMR. However, the study was uncontrolled.

Another single-center retrospective clinical study reported on 40 kidney transplant recipients with cAMR who received tocilizumab at a median time of 34 days after cAMR diagnosis. Multiple patients had received earlier immunosuppressive therapies prior to tocilizumab including rituximab (*n* = 16), plasmapheresis (*n* = 8), high dose corticosteroids (*n* = 21), or anti-thymoglobulin (*n* = 2), with only seven patients receiving tocilizumab as first-line treatment. Tocilizumab therapy was associated with stable eGFR (43 ± 17 ml/min/1.73 m^2^ at initiation; 45.3 ± 15 ml/min/1.73 m^2^ at month 6; 41.6 ± 17 ml/min/1.73 m^2^ at month 12), proteinuria (1.0 ± 0.9 g/l at initiation; 0.8 ± 1.1 g/l at month 6; 0.9 ± 1.1 g/l at month 12), and histopathology (glomerulitis, peritubular capillaritis, and interstitial fibrosis/tubular atrophy scores) showed no difference in patients who did not lose their allograft [27]. Another single-center retrospective analysis evaluating cAMR patients receiving tocilizumab therapy as first-line therapeutic option did not observe a beneficial effect in the absence of DSA [28].

Overall, there is a lack of adequately controlled studies on the impact of tocilizumab on cAMR. Available evidence from a limited number of patients suggests that tocilizumab treatment may help stabilize kidney injury when used as a first-line therapy, and potentially offer benefits as rescue therapy for some patients after other regimens for cAMR have failed. However, it is unclear whether such beneficial effects are superior to conventional therapies as there is a lack of direct comparison studies.

A small (*n* = 50), investigator-initiated ongoing phase 3 randomized controlled trial (RCT) is assessing the efficiency and safety of tocilizumab (NCT04561986, INTERCEPT) for cAMR in kidney transplant recipients. It is evaluating standard of care plus tocilizumab vs standard of care, with a primary endpoint of change from baseline in eGFR at 24 months and is expected to be completed in 2028. Additionally, a small (*n* = 18) phase 2 trial (RAIPONS, NCT04779957) is evaluating the safety of tocilizumab to reduce allo-sensitization post allograft nephrectomy.

#### Clazakizumab

Clazakizumab is a humanized monoclonal antibody against IL-6 with high affinity and a long half-life of ∼30 days, which allows monthly or bimonthly injections. It has been well-studied in the management of rheumatoid arthritis and psoriatic arthritis, although not yet approved for clinical use [29, 30]. It is also being developed for cardiovascular protection in patients with kidney failure undergoing hemodialysis (NCT05485961).

In a phase I/II open-label clinical trial investigating clazakizumab for cAMR, 10 adults with biopsy proven cAMR and transplant glomerulopathy with positive DSA were treated monthly with subcutaneous injections of 25 mg clazakizumab and underwent protocol biopsy at month 6. Clazakizumab therapy was associated with stable eGFR (41.90 ± 12.09 ml/min at 0 M vs. 38.86 ± 10.42 ml/min at 12 M, *P* = .60) and non-statistically significant numerical reductions in mean DSA (6.40 ± 3.31 historical vs 3.43 ± 4.58 at 12 M, *P* = .14) and CRP (1.11 ± 1.25 at 0 M vs 0.43 ± 0.17 at 12 M, *P* = .31) [31]. In a more recent phase II study, evaluating use of clazakizumab, involved patients with treatment-resistant cAMR of kidney allografts who received monthly 25 mg subcutaneous clazakizumab injections for 12 months, as in the previous study. Those who were stable at 12 months continued the study under a “long-term extension”; receiving clazakizumab injections longer than a 2.5-year period. Results revealed significantly declined mean eGFR values (52.8 ± 14.6 to 38.11 ± 12.23 ml/min per 1.73 m^2^, *P* = .03) from −24 to 0 months; on the other hand, stabilization of eGFRs at (41.6 ± 14.2 and 38.1 ± 20.3 ml/min per 1.73 m^2^) were observed at 12 and 24 months, respectively. DSA levels were also reduced in most of the patients as well as reductions in C4d scores. Minimal adverse events were encountered throughout the study, with only two graft losses in patients who discontinued the therapy early on the course [32].

A phase 2 pilot randomized placebo-controlled clinical trial evaluated the safety and tolerability as well as the efficacy of clazakizumab in 20 patients experiencing late with late-active or chronic active AMR (>1 year post-transplantation, median 10.6 years) and eGFR over 30 ml/min/1.73 m^2^. Participants received clazakizumab (25 mg every 4 weeks) or placebo for 3 months and this was followed by an open-label extension of clazakizumab for all participants for 40 weeks. Maintenance immunosuppression regimens were either calcineurin inhibitor or mTOR inhibitor-based triple regimens (*n* = 18) or dual regimens without corticosteroids (*n* = 2). Initial findings on efficacy indicated a potential positive impact of clazakizumab on reducing AMR activity and its progression. eGFR slopes were slower initially on clazakizumab than on placebo [−0.96; 95% confidence interval (95% CI), −1.96 to 0.03 versus −2.43; 95% CI, −3.40 to −1.46 ml/min per 1.73 m^2^ per month, respectively, *P* = .04] and they slowed down in placebo patients after switching to clazakizumab (*P* < .001). Clazakizumab decreased DSA titers over a 12-week clinical trial period (*P* = .035) with further decline in 40-week extension period (*P* < .001). Despite the lack of improvement in rejection-related molecular or morphological scores in biopsies at week 11, significant improvement in “all rejection” scores (*P* = .037) and molecular scores of AMR (*P* = .020) were observed at week 51 without any difference in T-cell mediated rejection scores at any time point. Moreover, C4d deposits disappeared in five patients (27.8%) along with total resolution of AMR activity in four patients (22.2%). Nonetheless, tubular atrophy and interstitial fibrosis increased significantly through weeks 11 to 51 potentially implicating the presence of ongoing tissue insult [33]. Long-term clazakizumab use was not associated with a compensatory increase in free circulating IL-6 and stopping clazakizumab did not result in rebound severity of cAMR [10]. However, a safety signal emerged for serious infection (occurring in 26% of participants in the open-label extension) and serious diverticulitis led to stopping therapy in 10% of participants.

IMAGINE (NCT03744910) is a double-blind, placebo-controlled phase 3 trial that expected to assess the safety and efficacy of clazakizumab in 350 kidney transplant recipients diagnosed with cAMR with a primary endpoint of change from baseline in eGFR at 24 months [34]. However, it was terminated in 2024 after recruiting 194 participants for lack of efficacy/futility at interim analysis [35, 36]. Overall, despite promising phase 2 trial results, clazakizumab is no longer in clinical development for cAMR.

## FUTURE PERSPECTIVES

cAMR is a troubling condition for kidney transplant recipients with limited therapeutic alternatives. However, it is unlikely that targeting IL-6 will offer an optimal alternative, given the futility results of the large phase 3 RCT assessing clazakizumab for cAMR. Even though the exact causes of failure of IL-6 targeting therapeutic options in the management of AMR are unclear, the potential hypotheses include (i) use of IL-6 targeting therapeutic options as a rescue therapy among patients who had already developed chronic and potentially irreversible histopathological alterations including transplant glomerulopathy; (ii) the lack of standardized therapeutic protocols used in the management of AMR leading to significant variations among clinical studies; (iii) potential concerns regarding the adverse effects including infectious and cardiovascular complications; and (iv) limited knowledge regarding the upregulation of regulatory T cells with IL-6 targeting therapies. While tocilizumab continues in clinical development, the smaller size of its phase 3 trial, the lack of a placebo group and the nearly 4-year timeline for completion makes it unlikely that IL-6 targeting will be routinely used to treat AMR in the foreseeable future; however, the role of tocilizumab in achieving kidney function stabilization should be noted as a promising aspect. Further, clinical development of IL-6 targeting therapies continue for kidney failure and CKD. Both clazakizumab and ziltivekimab decrease inflammation and improve anemia this context and are in clinical development for cardiovascular protection in CKD and kidney failure [6, 7]. A phase 3 trial of ziltivekimab (ZEUS, NCT05021835) for cardiovascular protection in CKD is expected to be completed by 2026 and another of clazakizumab (NCT05485961) in kidney failure in 2028. Both are enrolling thousands of patients and eventual approval of IL-6 targeting therapies for cardiovascular protection in CKD or kidney failure is expected to be followed by their assessment for this purpose in kidney transplantation.

## Data Availability

No new data were generated or analyzed in support of this research.
